# Combination phenylbutyrate/gemcitabine therapy effectively inhibits *in vitro *and *in vivo *growth of NSCLC by intrinsic apoptotic pathways

**DOI:** 10.1186/1477-3163-5-25

**Published:** 2006-11-23

**Authors:** Bodo Schniewind, Kirsten Heintz, Roland Kurdow, Ole Ammerpohl, Anna Trauzold, Doris Emme, Peter Dohrmann, Holger Kalthoff

**Affiliations:** 1 Hospital for General and Thoracic Surgery, Schleswig-Holstein University Hospitals, Campus Kiel, Arnold-Heller-Str 7, Kiel, Germany; 2 Molecular Oncology Section, Schleswig-Holstein University Hospitals, Campus Kiel, Arnold-Heller-Str 7, Kiel, Germany

## Abstract

**Background:**

Standard chemotherapy protocols in NSCLC are of limited clinical benefit. Histone deacetylase (HDAC) inhibitors represent a new strategy in human cancer therapy. In this study the combination of the HDAC inhibitor phenylbutyrate (PB) and the nucleoside analogue gemcitabine (GEM) was evaluated and the mechanisms underlying increased cell death were analyzed.

**Methods:**

Dose escalation studies evaluating the cytotoxicity of PB (0.01–100 mM), GEM (0.01–100 μg/ml) and a combination of the two were performed on two NSCLC cell lines (BEN and KNS62). Apoptotic cell death was quantified. The involvement of caspase-dependent cell death and MAP-kinase activation was analyzed. Additionally, mitochondrial damage was determined. In an orthotopic animal model the combined effect of PB and GEM on therapy was analyzed.

**Results:**

Applied as a single drug both GEM and PB revealed limited potential to induce apoptosis in KNS62 and Ben cells. Combination therapy was 50–80% (p = 0.012) more effective than either agent alone. On the caspase level, combination therapy significantly increased cleavage of the pro-forms compared to single chemotherapy. The broad spectrum caspase-inhibitor zVAD was able to inhibit caspase cleavage completely, but reduced the frequency of apoptotic cells only by 30%. Combination therapy significantly increased changes in MTP and the release of cyto-c, AIF and Smac/Diabolo into the cytoplasm. Furthermore, the inhibitors of apoptosis c-IAP1 and c-IAP2 were downregulated and it was shown that in combination therapy JNK activation contributed significantly to induction of apoptosis. The size of the primary tumors growing orthotopically in SCID mice treated for 4 weeks with GEM and PB was significantly reduced (2.2–2.7 fold) compared to GEM therapy alone. The Ki-67 (KNS62: p = 0.015; Ben: p = 0.093) and topoisomerase IIα (KNS62: p = 0.008; Ben: p = 0.064) proliferation indices were clearly reduced in tumors treated by combination therapy, whereas the apoptotic index was comparably low in all groups.

**Conclusion:**

Therapy combining GEM and the HDAC inhibitor PB initiates a spectrum of apoptosis-inducing mitochondrial and further JNK-dependent events, thereby overcoming the therapeutic resistance of NSCLC tumor cells. *In vivo*, the combination therapy substantially reduced tumor cell proliferation, suggesting that the well tolerated PB is a useful supplemental therapeutic agent in NSCLC.

## Background

Lung cancer is the leading cause of cancer related death world wide [1]. Only a minority of patients are suitable for potentially curative surgical intervention [2]. The majority of patients are managed with palliative therapy regimes based primarily on chemotherapy [3]. An increasing number of patients are being treated with neoadjuvant or adjuvant chemotherapy/radiotherapy based therapeutic strategies [4]. However, the effectiveness of such strategies is still very limited in terms of prolonging survival, and symptom relief and improving the quality of life remain the fundamental effects of current regimes [5].

Gemcitabine (GEM) is frequently applied in a combination therapy regime in patients with advanced lung cancer [6]. GEM enters the cells via a nucleoside transport system and is subsequently phosphorylated to inhibit ribonucleotide reductase (RNR) and to compete with dCTP for incorporation into DNA [7]. Like other nucleoside analogues, GEM is able to induce apoptosis in NSCLC cells. However, the clinical effectiveness in the treatment of lung cancer is often insignificant, and the major obstacle is that cancer cells exert substantial resistance towards chemotherapy induced apoptosis, which considerably limits the response to therapy [8,9].

Histone deacetylase (HDAC) inhibitors, including phenylbutyrate (PB), induce histone hyperacetylation, which alters the expression of numerous genes by interfering with chromatin structure [10]. This is associated with the induction of apoptosis, differentiation and the inhibition of proliferation in various solid and hematologic tumors, including lung cancer [11-13]. However, the clinical benefit of PB treatment alone in advanced malignancies was limited [14-16], although PB demonstrated a low toxicity profile. Nevertheless, PB has been FDA-approved for inborn urea cycle disorders and has a very favorable side-effect profile.

We recently demonstrated that gemcitabine induces apoptosis in lung cancer cell lines by recruiting caspases, mitogen-activated protein kinases (MAPK) and mitochondria triggered (intrinsic) apoptotic signaling [8]. However, the induction of apoptosis was profoundly blocked *in vitro *as well as *in vivo *by the strong apoptotic resistance of the tumor cells on the level of the mitochondria.

Here we report that PB and GEM in combination have a potent effect on cytotoxicity in NSCLC cancer cell lines. The rational for combining these agents was that HDAC inhibitors had been demonstrated to regulate the expression of multiple apoptotic mediators and induce mitochondria-dependent apoptosis in various malignant tumor cells, such as melanoma cells, osteosarcoma cells and leukaemia cells [17-19]. Additionally, Maggio et al. suggested that MAPK are involved in HDAC inhibitor induced apoptosis [19]. Here, we show that key events in mitochondria triggered apoptosis are stimulated by combination therapy, activation of MAPK is enhanced and inhibitors of apoptosis are down-regulated, resulting in potent tumor growth inhibition *in vitro *as well as *in vivo *in orthotopic tumor models.

## Methods

### Cell lines and culture conditions

The human lung cancer cell lines (KNS62 and Ben) have been described previously [8]. Non-genetically engineered cells were routinely maintained in RMPI 1640 supplemented with 10% FCS, 2 mM glutamine and 1 mM sodium pyruvate (Life Technologies, Inc., Eggenstein, Germany) without penicillin or streptomycin. All cells were kept in a humidified atmosphere containing 5% CO_2 _at 37°C.

### Immunohistochemical analysis

Resected orthotopically growing tumors were immediately frozen in liquid nitrogen. Five μm thick frozen tissue sections were cut from the tumor and fixed for 10 min in acetone. Immunohistochemical assessment of tumor proliferation was performed by the using monoclonal mouse antibody MIB-1 (Dianova, Hamburg, Germany) against the nuclear antigen Ki-67 and the monoclonal mouse antibody Ki-S4 against topoisomerase IIα (kindly provided by Prof. Parwaresch, Dept. of Hematopathology and Lymph Node Registry, Kiel, Germany). Immunolabeling with the specific antibody was evaluated by counting 200 tumor cells in 3 different "hot spots" in each cryosection at high-power magnification (× 400). Counting was done by 2 independent observers. The labeling indices were calculated as percentage of positive tumor cells. The mean values and standard deviations are based on 3 animals from each group. From each tumor bearing animal 3 cryosections were taken for analysis.

For staining of intratumoral vascular endothelium, cryosections were stained bwith they monoclonal rat anti-mouse MEC13.3 (BD Pharmingen, Hamburg, Germany) against CD31 (PECAM-1). The APAAP method was used for detection [20]. Microvessel density (MVD) was calculated according to Weidner et al. [21]. Briefly, areas of elevated vascular density were identified and subsequently the microvessel entities per optical field (× 200 magnification) were counted in 5 different areas of each tumor. Statistical mean values, SD and p values (t test) were calculated.

### Immunological reagents

Mouse anti-caspase 8 antibodies (clone 5F7) were obtained from Upstate Biotechnology (Eching, Germany). Anti-PARP (clone C-2-10) was obtained from Calbiochem (Bad Solden, Germany), anti-β-actin (clone AC-15) from Sigma, and anti-cytochrome c (clone 7H8.2C12) from Pharmingen (Hamburg, Germany). Rabbit polyclonal antibodies against Bcl-xL were obtained from Pharmingen, anti-Bid from R&D Systems (Wiesbaden, Germany), anti-caspase 9 from Cell Signaling (Berlin, Germany), antibodies to JNK, phosho-JNK, c-Jun and phosphor-c-Jun from Cell Signaling. Peroxidase-conjugated anti-rabbit IgG and anti-mouse IgG were obtained from Amersham (Brunswick, Germany). Rabbit polyclonal anti-cIAP1 H-83, and rabbit polyclonal anti-cIAP2 H-85 antibodies were purchased from Santa Cruz (Heidelberg, Germany). Rabbit monoclonal anti-survivin and anti-XIAP were obtained from Cell Signaling.

### Apoptosis assay

The NSCLC cancer cell line KNS 62 was seeded at a density of 1 × 10^4 ^cells/well into 96-well flat-bottom microtiter plates, allowed to adhere overnight and labeled with ^3^H-thymidine (370 kBq/μl; Amersham, Brunswick, Germany) for 3 h. Subsequently, the cells were washed with phosphate-buffered saline (PBS) and incubated with various concentrations of gemcitabine (Lilly), phenylbutyrate or a combination of the two in normal growth medium for up to 72 h. The cells were lysed in 0.05% SDS for 30 min at 37°C to ensure complete release of genomic DNA and harvested by vacuum aspiration on glass-fiber filters. Dried filters were counted using a liquid scintillation counter (Wallac, Switzerland). The percentage of specific DNA fragmentation, indicative of apoptosis, was calculated as: percentage viability = (E/S) × 100, where E (experimental) is the counts per minute (cpm) of retained DNA in the presence of chemotherapy and S (spontaneous) is the cpm of retained DNA in the absence of chemotherapy [9]. Caspase 3 and caspase 8 activity was measured by immunoblotting of total cellular proteins and subsequent detection of caspase 3 and caspase 8 and cleavage of their substrates PARP and Bid. The broad caspase inhibitor zVAD-fmk was obtained from Biomol, Ltd. (Hamburg, Germany). The following inhibitors of different Mitogen-Activated Protein Kinases (MAPK) were employed: 1 μmol/L of SP600125 a JNK-specific inhibitor, 10 μmol/L of SB203580 a p38-specific inhibitor and 0.5 μmol/L of MEK1/2-inhibitor, all from Calbiochem (Bad Soden, Germany).

### Cell cycle analysis and apoptosis measurement

The cells were washed twice with PBS, trypsinized, pelleted, resuspended in PBS containing 5 mM EDTA and fixed by adding one volume of ethanol (Merck, Darmstadt, Germany). After Rnas -treatment (40 ng RNaseA/μl, Sigma-Aldrich, Taufkirchen, Germany) cells were pelleted, resuspended in PBS containing propidium iodide (200 μg/ml) and subjected to FACS analysis. Cell cytometry was conducted using a FACScan cell analyzer (Becton-Dickinson Bioscience, San Jose, CA, USA). WinMDI2.8 [22] was used for analyzing FACS data.

### Mitochondrial transmembrane potential

Mitochondrial integrity was determined by assessing the loss of the mitochondrial membrane potential Δψ_m _using an ApoAlert Mitochondrial Membrane Sensor Kit (Clontech, Heidelberg, Germany) followed by FACScan (Becton Dickinson) analysis.

### Orthotopic transplantation of tumor cells into immunodeficient mice

Female SCID-bg (C.B-17/IcrHsd-***Prkdc***^***scid ***^***Lyst***^***bg***^) mice (Harlan Winkelmann, Borchen, Germany) weighing 14 to 19 g were allowed to become acclimatized for 1 week in a sterile environment where bedding, food and water were autoclaved. Food and water were given *ad libitum*. Animal experiments and care were performed in accordance with the guidelines of the institutional authorities. The mice were anaesthetized by i.p. injection of a mixture of Midazolam 5.0 mg/kg (Roche, Grenzach-Wehyen, Germany), Fentanyl 0.05 mg/kg (Janssen-Cilag, Neuss; Germany) and Medetomidin 5.0 mg/kg (Pfizer, Karlsruhe, Germany). The orthotopic animal model was performed as previously published [23]. Briefly, after right lateral thoracotomy the lung was carefully exposed and a tumor cell suspension (1 × 10^6 ^cells in 30 μl Matrigel, Biosciences) was cautiously injected into the lung tissue. The thoracic wall and the skin were closed with a 6-0 running absorbable suture (Ethicon, Norderstedt, Germany). After completion of the surgical procedure anaesthesia was antagonized by s.c. injection of a mixture of Flumazenil 0.5 mg/kg (Roche, Grenzach-Wehyen, Germany), Naloxon 1.2 mg/kg (Curamed, Neuss; Germany) and Atipamezol 2.5 mg/kg (Pfizer, Karlsruhe, Germany). All mice were inspected daily for complications. Once orthotopic KNS62 and Ben tumors were established (7 d after orthotopic tumor cell injection), the mice were treated with 50 mg/kg GEM i.p. twice a week for 28 days, 300 mg/kg PB by subcutaneous infusion with Alzet osmotic minipumps (model 2002; Alzet, Cupertino, CA, USA) or by combination therapy. The mMinipumps were exchanged after 2 weeks. In the control group NaCl was administered instead of chemotherapy according to the gemcitabine scheme. The animals were sacrificed after 35 days and the tumors were resected. Tumor weight and tumor volume according to the formula of a rotational ellipsoid (V = length × height × width × 0.5236) were calculated. Resected tumors were bisected and cryo- and formalin-fixed for further investigations. The data were analyzed using SPSS for Windows (V 11.0, Chicago, IL, USA). The results are given as means ± SD. Differences in tumor volume between relevant subgroups were analysed and p values were calculated by Mann-Whitney U test. A global p value of less then 0.05 was considered to be statistically significant.

## Results

### Sensitivity of lung cancer cells to GEM and PB mediated apoptosis

**Figure 1:**
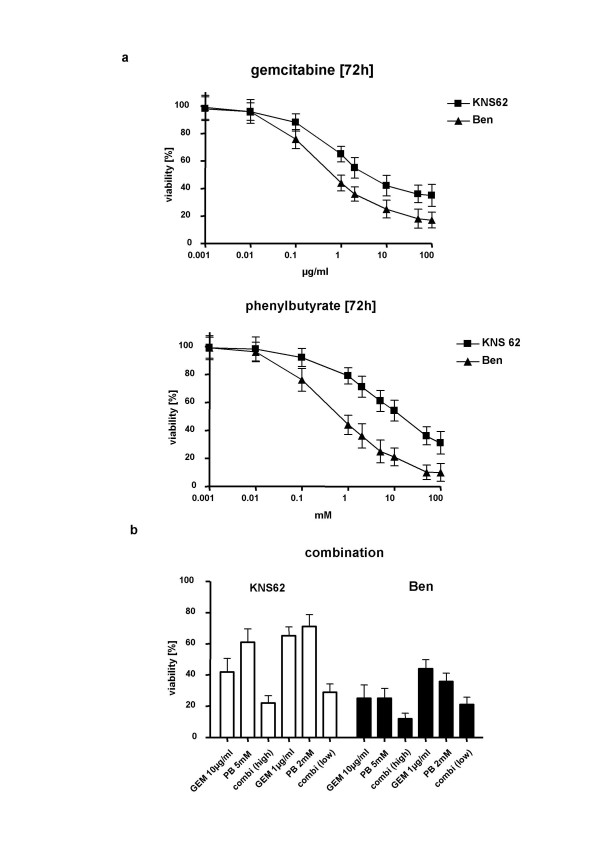
**DNA fragmentation of NSCLC cells by chemotherapy**. **A**. Dose-response curves for the NSCLC cell lines KNS62 and Ben towards gemcitabine (GEM); (upper panel) and phenylbutyrate (PB); (lower panel). Quantification of cellular DNA fragmentation by the JAM assay in lung carcinoma cells treated with different concentrations as indicated. **B**. DNA fragmentation by combination PB/GEM therapy with two different concentrations of combined chemotherapy. 100% viability = untreated control.

We analyzed the sensitivity of two different NSCLC cell lines (KNS62 and Ben) to increasing doses of GEM and PB. The cell lines underwent apoptosis in a dose-dependent manner, showing fragmentation of cellular DNA, though KNS62 was less sensitive than Ben to GEM and PB (Fig. 1, upper and middle panel). When GEM and PB were combined, either in high dosage (10 μg/ml GEM and 5 mM PB) or in low dosage (1 μg/ml GEM and 2 mM PB), the rate of viable cells was significantly decreased compared to single substance treatment. Remarkably, an effect exceeding the sum of single agent treatment was detectable in the KNS62 low dosage treatment group (Fig. 1, lower panel).

### Effect of GEM and PB combination treatment on apoptotic cell death

**Figure 2:**
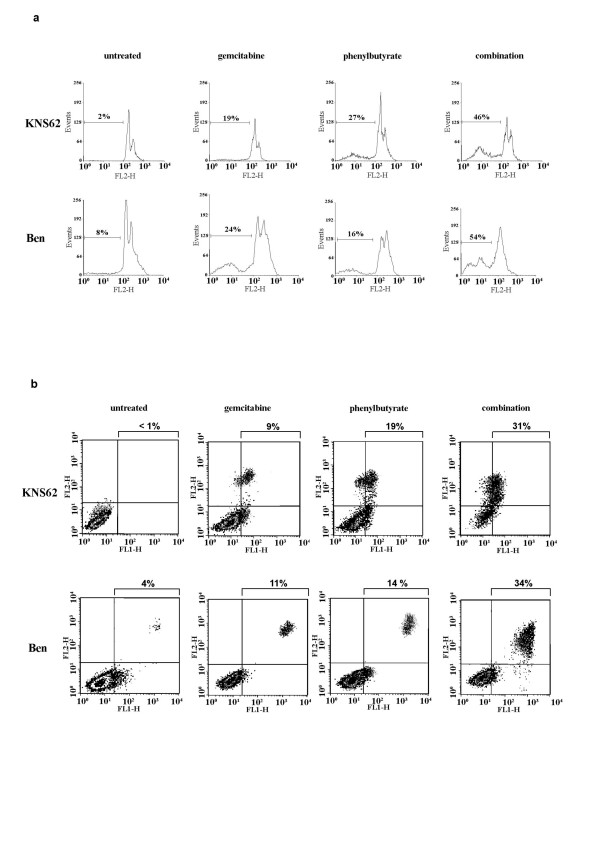
**Specification of apoptotic cell death induced by chemotherapy**. **A**. Quantification of apoptotic cells by PI-FACS cell cycle analysis for combined chemotherapy in KNS62 and Ben lung cancer cell lines. **B**. Quantification of apoptosis by Annexin-V/PI-FACS analyses. PI: FL2-H; Annexin: FL1-H

Various indicators of apoptotic cell death were investigated in KNS62 and Ben cells after treatment with GEM and PB in combination. PI-FACS analyses of the PI-stained cells focused especially on the sub-G1 cellular DNA fraction. The combination treatment revealed a significant increase in DNA in the sub-G1 fraction compared to gemcitabine treatment alone (Fig. 2, upper panel). After 72 h of combination therapy 46% of KNS62 cells and 54% of Ben cells were detectable in the sub-G1 cellular (apoptotic) fraction, compared to only 19% of KNS62 and 24% of Ben after treatment with gemcitabine alone. To quantify the early apoptotic phenotype Annexin-V/PI-FACS analyses were performed (Fig. 2, lower panel). As early apoptotic events Annexin-V-positive cells (right lower quadrant in the density dot plot) as well as PI-positive and Annexin-V-positive cells (right upper quadrant) were summarized. After combination therapy significantly more cells revealed early morphologic events of apoptosis (KNS62: 34%; Ben: 39%) than cells treated with gemcitabine alone (KNS62: 11%; Ben: 14%).

### Activation of caspases by combined chemotherapy

**Figure 3:**
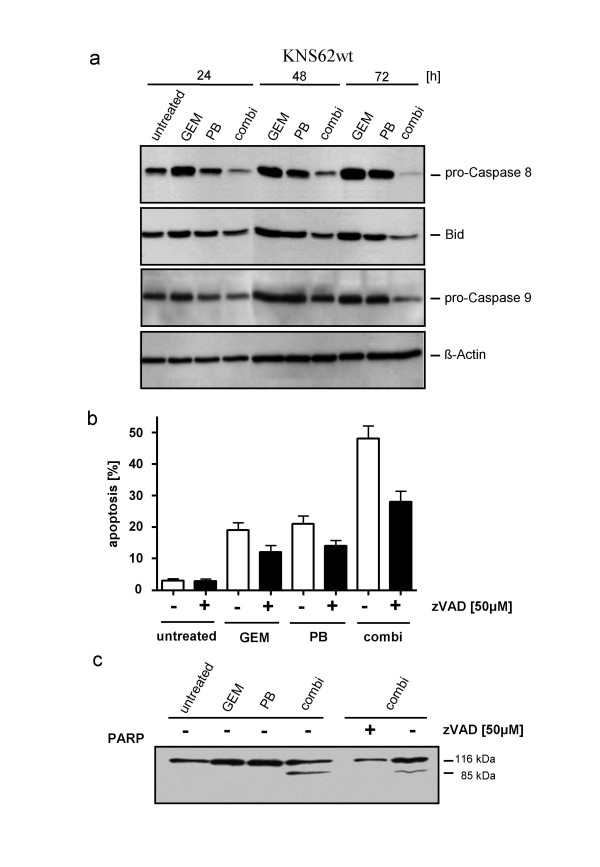
**Caspase-dependent apoptotic cell death**. Caspase activation in the lung cancer cell line KNS62 by combination chemotherapy. **A**. Detection of caspase-8, Bid, caspase-9 and PARP cleavage in KNS62 after the indicated treatment. **B**. Inhibition of apoptosis by broad caspase inhibitor zVAD after corresponding treatment. **C**. Inhibition of PARP cleavage zVAD after treatment.

The activation of key apoptotic proteins was investigated to evaluate the influence of GEM, PB and combination chemotherapy on apoptosis at the molecular level. In death receptor-mediated apoptosis, receptor activation is followed by cleavage of caspase-8 and its substrate BID, a BH3 domain-containing pro-apoptotic protein that subsequently becomes activated. Cleavage of caspase-8 and Bid was low in KNS62 cells after GEM and PB treatment alone, but significantly increased with combination therapy (Fig. 3a). Similarly, cells subjected to combination therapy exhibited clearly increased cleavage of caspase-9 in KNS62.

The dependence of chemotherapy-induced cell death on caspase-mediated apoptotic pathways was confirmed by the observation that the broad caspase-inhibitor zVAD prevented apoptosis-related DNA fragmentation and PARP cleavage in treated cells (Fig. 3b+c). However, DNA fragmentation was only partially inhibited, suggesting further mechanisms besides caspase-dependent apoptosis.

### Mitochondrial integrity after combined chemotherapy

**Figure 4:**
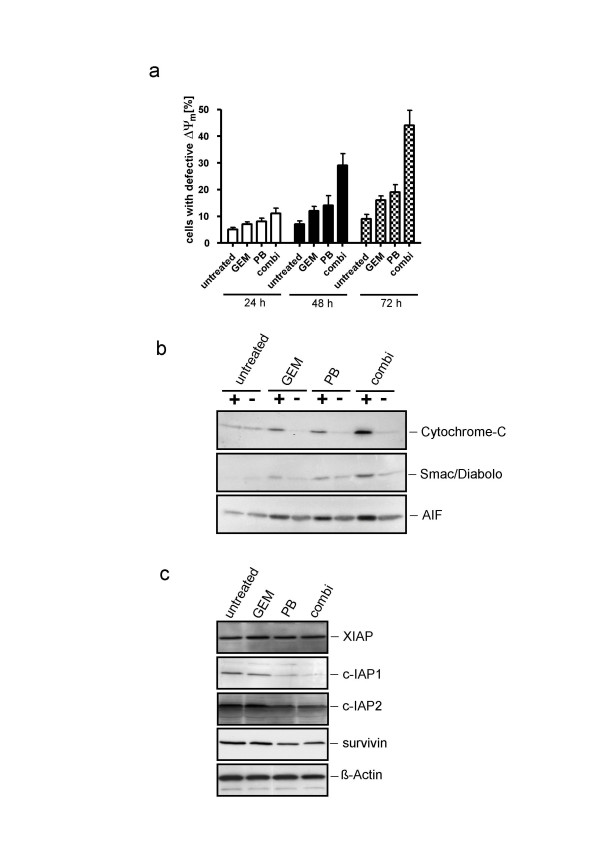
**Chemotherapy induced changes in mitochondrial stability**. **A**. Measurement of the loss of Δψm in KNS62 treated with indicated chemotherapy at different time points. Effect of combination chemotherapy on mitochondrial function. **B**. Detection of cytochrome-c, Smac/Diabolo and AIF release into the cytoplasma after 48 h of therapy. **C**. Expression levels of the IAP members XIAP, c-IAP1, c-IAP2 and survivin after 48 h of treatment.

The involvement of mitochondria in chemotherapy-mediated apoptosis was determined by assessing mitochondrial integrity. After 24 h of combination chemotherapy only 11% of KNS62 cells exhibited a loss of Δψm, compared to 7% in the gemcitabine group and 8% in the phenylbutyrate group. This difference increased over time from 29% (48 h) and 44% (72 h) of cells with defective Δψm in the combination group compared with 12%/16% (48/72 h) for gemcitabine and 14%/19% (48/72 h) for phenylbutyrate (Fig. 4a).

These results were confirmed by the demonstration of cytochrome-c, Smac/Diabolo and AIF release from mitochondria into the cytosol, as detected by Western blot analyses of cytosolic proteins. In the cytosolic fractions of combination chemotherapy-exposed KNS62 cells there was a significantly higher release of cytochrome-c, Smac/Diabolo and AIF into the cytosole compared to single agent (gemcitabine or phenylbutyrate) therapy (Fig. 4b). An analysis of protein expression levels of various family members of the inhibitors of apoptosis proteins (IAP) revealed that especially c-IAP1 and c-IAP2 were significantly down regulated by PB and combination therapy, whereas XIAP remained stable and survivin showed only moderate regulation (Fig. 4c).

### JNK regulates combination chemotherapy induced apoptosis

**Figure 5:**
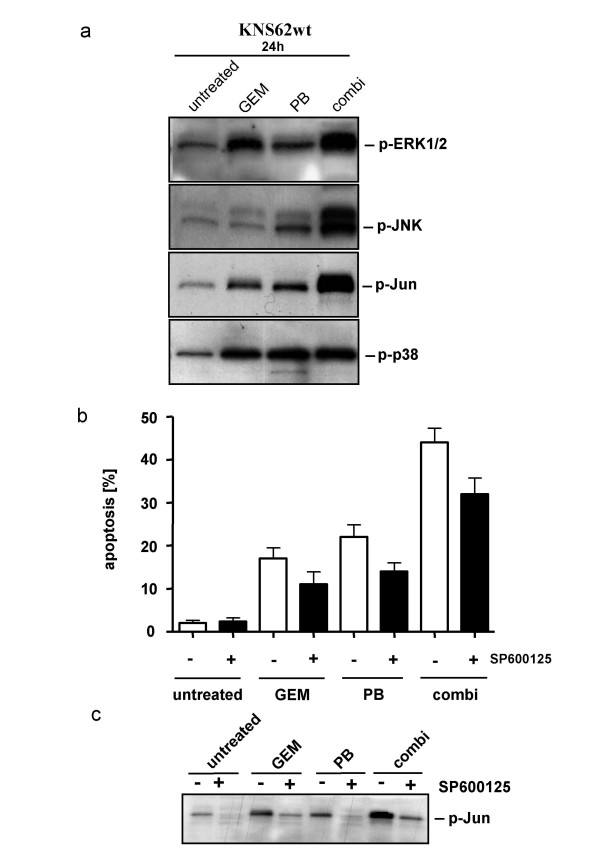
**Involvement of MAPK activation in chemotherapy induced apoptosis**. **A**. Phosphorylation of the MAPK members ERK1/2, JNK and its target c-Jun and p38 by the indicated treatment. **B**. Inhibition of apoptosis by the JNK inhibitor SP600125. **C**. Inhibition of phosphorylation of c-Jun by JNK-inhibitor.

Since mitogen-activated protein kinases (MAPK) have been determined to be substantially involved in controlling chemotherapy-induced apoptosis [24,25], we investigated the involvement of MAPK in GEM and PB combination therapy-induced apoptosis. While treatment of KNS62 with either GEM or PB induces phosphorylation of ERK1/2, p38, JNK and its target c-Jun, combination therapy amplifies this effect substantially (Figure 5a). The overall level of these proteins (ERK1/2, p38, JNK and c-Jun) remaiedn unchanged (data not shown). The impact of activation of different MAP-Kinases on apoptosis was tested by co-incubation of specific inhibitors. Only specific blocking of p-JNK significantly inhibited the induction of apoptosis by chemotherapy (Figure 5b), whereas the level of phosphorylated c-Jun as the target of activated JNK was effectively decreased by the JNK inhibitor SP600125 (Fig. 5c).

### Orthotopic growth of NSCLC tumors in SCID mice treated with GEM and PB chemotherapy

**Figure 6:**
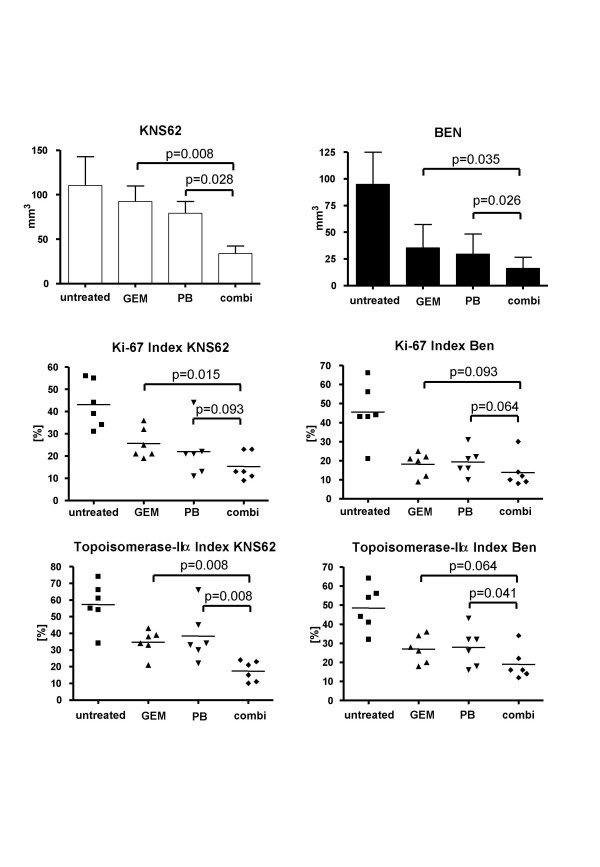
**Inhibition of *in vivo *tumor growth by chemotherapy**. **A**. Tumor volume of orthotopically growing KNS62 and Ben tumours treated with gemcitabine, phenylbutyrate or combination chemotherapy for 4 weeks. **B**. Ki-67 labeling indices of orthotopically growing tumors. **C**. Topoisomerase IIα labeling indices of orthotopically growing tumors.

The effect of gemcitabine and phenylbutyrate on *in vivo *tumor growth was investigated in an orthotopic SCID mouse model. Each group comprised six animals. In untreated animals, KNS62 the mean tumor size was 110 mm^3 ^(SD: 32.6) compared to 92.5 mm^3 ^(SD: 42.2) in the GEM group, 79.3 mm^3 ^(SD: 37.0) in the PB group and 33.8 mm^3 ^(SD: 24.3) in the combination group. The tumor size was significantly smaller in the combination group compared to GEM (p = 0.008) or PB (p = 0.028) chemotherapy alone (Fig. 6a).

In orthotopically growing Ben tumors the mean tumor size in the untreated group was 95 mm^3 ^(SD: 30), in the GEM group 36.6 mm^3 ^(SD: 21.6), in the PB group 29.7 mm^3 ^(SD: 18.8) and in the combination therapy group 16.2 mm^3 ^(SD: 10.3). Like in the KNS62 orthotopic model in the Ben tumors were significantly smaller in the combination therapy group compared to GEM (p = 0.035) and PB (p = 0.026) (Fig. 6a).

The analysis of the proliferation activity of orthotopically growing tumors by means of Ki-67 and topoisomerase IIα staining indices revealed significant inhibition of proliferation in both combination therapy groups (**KI-67**: KNS62: GEM = 25.7% vs. combi = 15.3%; Ben: GEM = 18.2% vs. combi = 13.8%; **topoisomerase IIα**: KNS62: GEM= 34.7% vs. combi = 17.3%; Ben: GEM = 27% vs. combi = 19%) compared to untreated animals or animals with single agent therapy (Fig. 6b+c).

The rate of apoptotic cells (staining of cytokeratin 18 fragments with M30 antibody and phosphorylation of histone H2B with anti p-H2B) was only slightly elevated (data not shown). The microvessel density (MVD; anti-CD31 positivity) was also only slightly lower in the combination group (data not shown).

## Discussion

NSCLC is still associated with a very poor prognosis, and the effectiveness of current chemotherapy protocols is still very limited in terms of prolonging survival [5]. However, new strategies, such as the inhibition of deacetylation of histones, have been developed to overcome the resistance of tumor cells to chemotherapy. Acetylation of histones induces changes in the chromatin structure and increases the accessibility of the DNA, leading to gene activation. On the other hand deacetylation by histone deacetylase (HDAC) inactivates gene expression. This was specified as epigenetic modification of gene expression [26]. Such a strategy might address deregulated genes in lung cancer tumor tissue that are responsible for tumor progression and therapy resistance [27].

A few studies have demonstrated anti-tumoral effects of various HDAC inhibtors even in phase II clinical trials, although the effectiveness as single agent therapy was limited and our understanding of the underlying mechanisms remain superficial [28].

The HDAC inhibitor PB belongs to the family of short fatty acids and is used for treatment of inborn defects of the urea cycle without major side effects [29]. The dosages administered in the animal models in this study were comparable to those applied in the clinical setting; therefore PB qualifies for a rapid transfer to clinical testing. We demonstrated that PB effectively increased GEM induced apoptosis in NSCLC cell cancer cell lines both *in vitro *and *in vivo*. In this context several studies have demonstrated in NSCLC that especially resistance to intrinsic pathway mediated apoptosis is associated with strong resistance to chemotherapy, especially on the level of ineffective caspase activation [8,9]. This is in line with other studies showing that in leukemia, prostate cancer and colon cancer the combination of conventional chemotherapy with HDAC inhibitors was able to enhance the effectiveness of therapy substantially [12,30-32].

Several authors have identified numerous differentially expressed genes in NSCLC compared to normal tissue that might be relevant for apoptotic resistance to chemotherapy[33,34]. We investigated the activation of several central apoptosis regulators, such as caspase-8 and its substrate Bid, caspase-9 and caspase-3, along with crucial biochemical parameters such as mitochondrial integrity and release of cytochrome-c, Smac/Diabolo and AIF into the cytoplasm. By employing PB, we addressed the aberrant expression of various genes simultaneously and not only the expression of one or few specific genes. Whereby apoptosis controlling pathways might be reactivated [29]. In this context we were able to show that combination therapy substantially increased the activation of the above mentioned key players in apoptotic cell death compared to single agent chemotherapy. Especially the blockage of these key activators contributes to chemotherapy resistance in lung cancer [35]. Therefore, the pro-apoptotic signaling of the HDAC inhibitor PB and GEM converge and substantially improve the impact on tumor growth suppression.

In the context of enhanced mitochondria triggered cell death due to disrupted mitochondrial transmembrane potential we detected the release of cytochrome-c, AIF and Smac/Diabolo into the cytoplasm, decreased levels of anti-apoptotoc c-IAP1 and c-IAP2 but unchanged levels of XIAP. These results are in accordance with the results of Yang at al. 2004, who identified Smac/Diabolo as a key molecule for selectively reducing protein levels of c-IAPs and in this way contributing to enhanced apoptosis.

Noteworthy in this regard is the release of the caspase-independent cell death effector AIF into the cytoplasm, which likely helps to explain why in this study combined chemotherapy induced apotosis was partially inhibited by the broad spectrum caspase-inhibitor zVAD. This is supported by several studies showing that AIF significantly contributes to caspase-independent cell death [36,37].

Our further analysis of the PB-mediated sensitizing effects demonstrated that PB significantly enhanced the gemcitabine-mediated activation of JNK. Inhibition of JNK activity by the JNK inhibitor SB600125 partially reduced chemotherapy-mediated apoptosis. This finding is in line with a recent study demonstrating the relevance of the JNK pathway for *in vitro *apoptosis induction due to single drug PB treatment in lung carcinoma cells [24]. In our model system inhibition of the JNK pathway particularly decreased the gemcitabine dependent cell death.

The in vitro results suggest that apoptosis is the predominant mechanism for increasing tumor cell sensitivity towards gemcitabine and phenylbutyrate combination chemotherapy. However, in *in vivo *tumors analyzed in the combination group the rate of apoptotic cells (staining of cytokeratin 18 fragments with M30 antibody and phosphorylation of histone H2B with anti p-H2B) was only slightly elevated (data not shown). This might be explained by the long treatment period, during which the majority of the apoptotic cells were already eliminated. The microvessel density (MVD; anti-CD31 positivity) was also only slightly lower in the combination group. However, suppression of angiogenesis by HDAC inhibitors might have an impact on tumor growth inhibition, as previously demonstrated in a prostate cancer model [38]. In the *in vivo *model the main effects on combination therapy were evident on the level of significantly reduced cell proliferation, as demonstrated by strongly reduced staining indices for KI-67 and topoisomerase IIα.

## Conclusion

In summary, these results demonstrate that the combination of gemcitabine and the HDAC inhibitor phenylbutyrate is an effective treatment regime for NSCLC by improved activation of caspase-dependent, mitochondria transmembrane stability mediated and JNK-activated apoptotic cell death. These *in vitro *findings together with two clinically relevant tumor model systems provide strong evidence that the well tolerated drug PB might be a promising supplemental therapeutic agent for the treatment of NSCLC and should be further evaluated in a clinical setting.

## Competing interests

The author(s) declare that they have no competing interests.

## Authors' contributions

BS designed the study and participated in all experiments. KH also participated in all experiments. RK substantially participated in the animal experiments. OA and AT performed the immunoassys and FACS analyses. PD participated in the design and coordination of the experiments. HK contributed to the conception of the study, participated in its design and helped to draft the manuscript. All authors read and approved the final manuscript.
